# Antiseptics among the Ancient Greeks

**Published:** 1890-11

**Authors:** 


### Antiseptics among the Ancient Greeks.

Prof. Andreas Anagostakis, of Athens, gives in the *Deutsche Medicinische Wochenschrift* some interesting facts in reference to the employment of antiseptic measures among the ancient Greeks. Hippocrates and Galen were aware that an unclean condition of wounds retarded healing. They were also well acquainted with the fact that by thorough hæmostosis, suture and the employment of antiseptic measures, infection of wounds might be prevented. Hippocrates warned his disciples against the use of moist dressings on account of the danger of suppuration, and forbade the employment of drugs before the wound was dry. Above all, says Galen, avoid dirt, as it prevents healing. The ancient Greeks boiled their water before applying it to wounds. Sponges were avoided, and charpie recommended in their stead, which was to be destroyed after use. One of the principal antiseptic substances then in use was wine, which was usually heated before using, and with which, according to Hippocrates, all wounds were to be washed. Dressings dipped in wine were also applied to the wound. Salt was in very general use, either in solution or in the form of sea-water. The solutions were rendered aseptic by boiling. Sulphate of copper was relied upon as an antiseptic for foul wounds, and was also put into use as a hæmostatic. Tar was highly prized for its antiseptic virtues, and was either applied in the form of a dressing or directly poured upon the wound. Besides these many aromatics and bitters were in daily usage, among which were thyme, rosin, asphaltum, etc., used as dressings, or in the form of plasters. Galen was acquainted with cat-gut, and advised the use of non-putrefying substances for sutures. Prof. Anagnostakis declares that all this was not empiricism, but an antiseptic method founded upon some knowledge of the priciples governing the healing of wounds.—*Druggist's Circular.*

